# Metalloprotease NleC Suppresses Host NF-κB/Inflammatory Responses by Cleaving p65 and Interfering with the p65/RPS3 Interaction

**DOI:** 10.1371/journal.ppat.1004705

**Published:** 2015-03-10

**Authors:** Andrea Hodgson, Eric M. Wier, Kai Fu, Xin Sun, Hongbing Yu, Wenxin Zheng, Ho Pan Sham, Kaitlin Johnson, Scott Bailey, Bruce A. Vallance, Fengyi Wan

**Affiliations:** 1 W. Harry Feinstone Department of Molecular Microbiology and Immunology, Bloomberg School of Public Health, Johns Hopkins University, Baltimore, Maryland, United States of America; 2 Department of Biochemistry and Molecular Biology, Bloomberg School of Public Health, Johns Hopkins University, Baltimore, Maryland, United States of America; 3 Division of Gastroenterology, Department of Pediatrics, BC’s Children’s Hospital and Child and Family Research Institute, Vancouver, British Columbia, Canada; 4 Department of Oncology, Sidney Kimmel Comprehensive Cancer Center, Johns Hopkins University, Baltimore, Maryland, United States of America; University of Toronto, CANADA

## Abstract

Attaching/Effacing (A/E) pathogens including enteropathogenic *Escherichia coli* (EPEC), enterohemorrhagic *E*. *coli* (EHEC) and the rodent equivalent *Citrobacter rodentium* are important causative agents of foodborne diseases. Upon infection, a myriad of virulence proteins (effectors) encoded by A/E pathogens are injected through their conserved type III secretion systems (T3SS) into host cells where they interfere with cell signaling cascades, in particular the nuclear factor kappaB (NF-κB) signaling pathway that orchestrates both innate and adaptive immune responses for host defense. Among the T3SS-secreted non-LEE-encoded (Nle) effectors, NleC, a metalloprotease, has been recently elucidated to modulate host NF-κB signaling by cleaving NF-κB Rel subunits. However, it remains elusive how NleC recognizes NF-κB Rel subunits and how the NleC-mediated cleavage impacts on host immune responses in infected cells and animals. In this study, we show that NleC specifically targets p65/RelA through an interaction with a unique N-terminal sequence in p65. NleC cleaves p65 in intestinal epithelial cells, albeit a small percentage of the molecule, to generate the p65^1–38^ fragment during *C*. *rodentium* infection in cultured cells. Moreover, the NleC-mediated p65 cleavage substantially affects the expression of a subset of NF-κB target genes encoding proinflammatory cytokines/chemokines, immune cell infiltration in the colon, and tissue injury in *C*. *rodentium*-infected mice. Mechanistically, the NleC cleavage-generated p65^1–38^ fragment interferes with the interaction between p65 and ribosomal protein S3 (RPS3), a ‘specifier’ subunit of NF-κB that confers a subset of proinflammatory gene transcription, which amplifies the effect of cleaving only a small percentage of p65 to modulate NF-κB-mediated gene expression. Thus, our results reveal a novel mechanism for A/E pathogens to specifically block NF-κB signaling and inflammatory responses by cleaving a small percentage of p65 and targeting the p65/RPS3 interaction in host cells, thus providing novel insights into the pathogenic mechanisms of foodborne diseases.

## Author Summary

The nuclear factor kappaB (NF-κB) signaling pathway is crucial for host defense, as it orchestrates both innate and adaptive immune responses. Beyond the best-studied Rel proteins (p65, RelB, c-Rel, p50 and p52), RPS3 has been recently identified as a “specifier” component of NF-κB, modulating the promoter selectivity and transcriptional specificity of NF-κB. In particular, the RPS3/p65-conferred signaling pathway was recently shown to play a critical role in host proinflammatory transcription and immune responses. Attaching and effacing (A/E) pathogens and others have acquired sophisticated mechanisms to modulate host NF-κB signaling pathways. We have found that NleC, a metalloprotease effector secreted by A/E pathogens, modulates host NF-κB signaling and inflammatory responses through a novel mechanism. NleC specifically recognizes and cleaves a small percentage of p65 and the generated N-terminal fragment of p65 interferes with the p65/RPS3 interaction, thereby amplifying the effect of cleaving only a small percentage of p65 molecules to selectively inhibit NF-κB gene expression. Our findings highlight a previously unappreciated mechanism through which pathogen-encoded proteases interfere with signaling cascades and inflammatory responses in host cells.

## Introduction

Foodborne diseases caused by enteric pathogens remain a significant and common health threat and an immense economic burden worldwide [1]. Among the causative agents of foodborne illness, diarrheagenic strains of *Escherichia coli* including enteropathogenic *E*. *coli* (EPEC) and enterohemorrhagic *E*. *coli* (EHEC), typically cause diarrhea, hemorrhagic colitis, and pediatric renal failure [2]. EPEC, EHEC, and the rodent-specific pathogen *Citrobacter rodentium* produce characteristic attaching/effacing (A/E) lesions on the host intestinal epithelium after they adhere to these cells [3]. These pathogens translocate a variety of virulence proteins (effectors), through a conserved type III secretion system (T3SS), into intestinal epithelial cells (IECs) to modulate host cell functions to the pathogen’s advantage [4,5]. An ever-expanding repertoire of T3SS secreted effectors, termed non-LEE-encoded (Nle) effectors, was recently identified in A/E pathogens [6,7,8,9,10]. The target proteins of Nle effectors in host cells have started to be identified [11,12,13,14,15,16,17,18,19,20]; however, it remains largely unknown how Nle effectors interfere with cell signaling cascades and dampen the immune responses in host cells. The recognition of pathogens by host sensors activates multiple signaling pathways to induce inflammatory responses and eradicate the pathogens [21]. Among those, the NF-κB signaling pathway is crucial for host defense, as it orchestrates both innate and adaptive immune responses [21]. On the other hand, A/E bacteria, like other successful pathogens, have acquired sophisticated mechanisms to modulate host NF-κB signaling pathways [22,23,24,25,26,27,28]. Not surprisingly, a handful of the Nle effector target proteins within host cells have been revealed to be NF-κB signaling molecules [11,12,13,14,15,16,17,18,29,30]. Notably, however the molecular mechanisms through which each of these Nle effectors modulate NF-κB signaling have not been fully elucidated [25,31].

Besides the well-defined Rel family proteins (RelA/p65, RelB, c-Rel, p50 and p52) [32], RPS3 and Src-associated substrate during mitosis of 68kDa (Sam68) were recently identified as “specifier” components of NF-κB [33], where they modulate the promoter selectivity and transcriptional specificity of NF-κB [34,35]. The nuclear translocation and “specifier” function of RPS3 have been revealed to be tightly regulated by NF-κB signaling cascades [18]. Specifically, RPS3 is found in the cytoplasmic p65-p50-IκBα inhibitory complex in resting cells [34]. External stimuli activate the IκB kinase (IKK) complex, of which IKKβ phosphorylates IκBα resulting in its subsequent ubiquitination and degradation. IκBα removal unmasks a nuclear localization sequence (NLS), which allows nuclear import of p65 and p50 [36]. Likewise, IKKβ phosphorylates RPS3 at serine 209 (Ser209), independently enhancing the RPS3-importin-α interaction for nuclear translocation. Once in the nucleus, RPS3 cooperates with p65 to target NF-κB to select promoters and to trans-activate those genes [18]. Of note, the significance of RPS3/NF-κB signaling pathway has been highlighted in an increasing number of pathophysiological conditions [17,18,34,37,38,39], particularly in host proinflammatory transcription and immune responses against enteric pathogen infections [17,18]. More specifically, the EHEC NleH1 effector inhibits the nuclear translocation of RPS3, but not p65, during NF-κB activation by tempering RPS3 Ser209 phosphorylation [17,18]. As a consequence, NleH1 reduces the transcription of select, but not all, NF-κB target genes; most of the NleH1-attenuated RPS3/NF-κB-dependent genes encode proinflammatory cytokines/chemokines [17,18]. In support of the critical role of RPS3 in the transcriptional selectivity of NF-κB genes, we recently demonstrated that modulating the RPS3/p65 interaction by ectopic expression of an N-terminal fragment (amino acids 21–186) of p65 attenuates RPS3 nuclear translocation, without affecting p65, thus selectively blocking a subset of specific NF-κB gene transcription [40].

NleC, a zinc-dependent protease effector conserved among A/E pathogens, was recently identified as one of the key effectors that dampen the innate immune response in host cells, particularly the production of inflammatory cytokines including interleukin-8 (IL-8) as well as others [16,20,30]. Mutagenesis of the consensus zinc metalloprotease motif _183_HEIIH_187_ abrogates the proteolytic activity of NleC and cleavage of host target proteins [16,29,30,41]. Although p300 [42], IκB [29], and the NF-κB Rel proteins p65 and p50 [16,29,30,41,42] have been reported as targets of NleC, it is largely understood that NleC cleaves and inactivates the NF-κB signaling pathway by primarily targeting the Rel proteins [16,29,30,41], in line with the known critical role of NF-κB in the transcription of inflammatory cytokine genes [43]. Previous studies have shown that ectopically expressed or T3SS-translocated NleC degrades p65, p50 and c-Rel, but not signal transducer and activator of transcription 1 (STAT1) or extracellular-signal-regulated kinases (ERKs), which indicates there may be cleavage specificity of NleC for the Rel subunits of NF-κB signaling pathway [30,41]. However, work by Yen *et al*. suggests that NleC could be specific for p65, as recombinant NleC could not digest p50 in cell lysates [16]. Moreover, two cleavage site(s) on p65 by NleC have been identified as between proline 10 and alanine 11 (P10/A11) [16] or cysteine 38 and glutamic acid 39 (C38/E39) [41,44,45], although none of these studies ruled out the other cleavage site experimentally. The detailed mechanisms on how NleC specifically recognizes and cleaves p65 remain poorly understood. Moreover, even though recombinant proteins and ectopic expression of NleC in cell lines were employed in previous studies, only a very small percentage of p65 was shown to be cleaved by NleC with a large portion of full-length p65 still present within the cells [16,41]. In contrast, it is puzzling that previous studies reported that NF-κB activity by luciferase assays and IL-8 production were markedly increased in HeLa cells infected with EPEC *ΔnleC* mutant, compared to wild-type EPEC [16,20,29,30,41,42].

Here we reveal that the N-terminus of p65 is specifically recognized by NleC, which is required for the subsequent NleC-mediated cleavage. Infection of mice with a *C*. *rodentium* mutant strain lacking NleC (*ΔnleC)* augmented the transcription of several proinflammatory cytokine genes including *Cxcl1*, *Cxcl2*, *Il1b*, *Ifng*, and *Il22*, and triggered more immune cell infiltration in the colon, compared to wild-type *C*. *rodentium* inoculation. Moreover, NleC primarily cleaves p65 at C38/E39 during *C*. *rodentium* infection, and we show that the generated p65^1–38^ fragment binds to RPS3 in NF-κB complexes and selectively retards the nuclear translocation of RPS3, but not p65. While only a small percentage of molecules are cleaved, the association between the p65^1–38^ fragment and RPS3 that interferes with RPS3/p65 interaction-mediated transcription amplifies the impact of NleC cleaving p65. Therefore our results reveal a novel mechanism by which A/E pathogens selectively dampen RPS3 signaling and ensure inhibition of the RPS3/NF-κB-dependent inflammatory responses in host cells.

## Results

### NleC specifically cleaves p65, but not other NF-κB subunits

**Fig 1:**
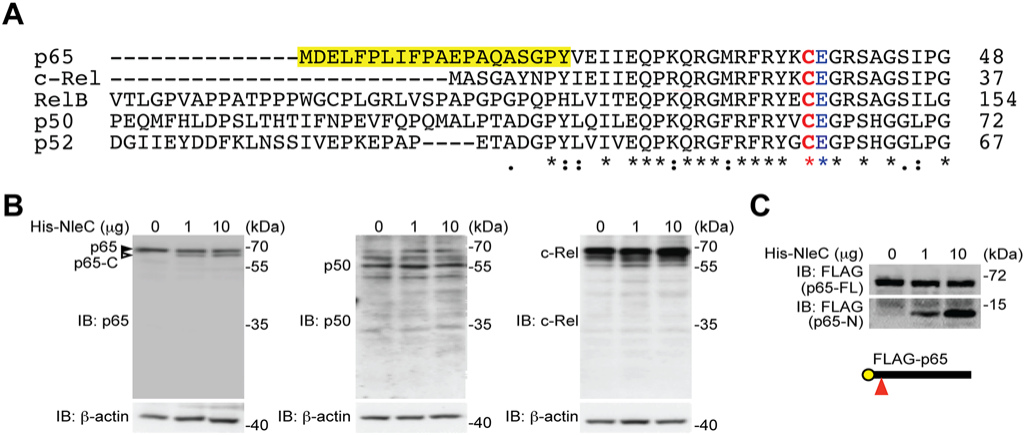
NleC specifically cleaves p65/RelA among the NF-κB Rel family proteins. **A**. Sequence alignment of the human Rel family proteins. The conserved Cys/Glu residues in their respective Rel domains and the unique N-terminal 20 residues in p65/RelA are highlighted. The numbers at right show the position in the amino-acid sequence of the last residues depicted. **B**. Whole cell lysates derived from HEK293T cells were incubated with indicated amount of His-NleC recombinant protein, followed by SDS/PAGE separation and immunoblotted for indicated proteins with antibodies specifically recognizing the C-termini of p65, p50 and c-Rel, respectively. The full-length p65 and cleaved p65 C-terminal fragment are indicated by filled and open triangles, respectively. **C**. Whole cell lysates derived from HEK293T cells expressing N-terminally FLAG-tagged p65 were incubated with indicated amount of His-NleC recombinant protein. The cleavage of FLAG-tagged p65 was immunoblotted with anti-FLAG antibody, following SDS/PAGE separation. The NleC cleavage site in p65 is indicated by a red triangle.

The T3SS effector NleC from various A/E pathogens has been proposed to dampen the NF-κB-mediated proinflammatory responses in host cells by functioning as a metalloprotease that cleaves NF-κB, albeit through poorly-defined mechanism(s) [16,20,29,30,41,42]. Moreover, Rel subunits p65 [16,20,29,30,41,42], p50 [29], and c-Rel [16,20,29,30,41,42] were proposed to be NleC target proteins. Alignment of the N-terminal sequences of both human and mouse Rel family proteins reveals that the best-characterized cleavage site, C38/E39 within the Rel homology domain, in p65 is also conserved among all human and mouse Rel proteins (Fig. 1A and S1 Fig), indicating that NleC could potentially cleave all Rel proteins. To clarify which Rel protein(s) are NleC targets, we incubated whole cell lysates derived from HEK293T cells with recombinant NleC protein and examined the cleavage of endogenous Rel proteins using antibodies that specifically recognize the C-terminus of each Rel subunit. Consistent with previous studies [16,20,29,30,41,42], a percentage of endogenous p65 was cleaved by NleC at the N-terminus thus generating a large C-terminal fragment (Fig. 1B), whereas a catalytically inactive mutant of NleC, *i*.*e*. NleC (H117Y) with histidine 117 in the HExxH motif replaced by tyrosine [16], failed to do so (S2 Fig). Moreover, NleC cleaved p65 to a similar extent in the presence and absence of tumor necrosis factor (TNF), a strong NF-κB stimulus (S3 Fig), indicating that the NleC-mediated partial cleavage of p65 is not due to protection by other p65-binding proteins in the cytoplasm. In contrast to p65, the cleavage of other endogenous Rel proteins was not detectable, even when incubated with overwhelming amount (10 μg) of NleC recombinant protein (Fig. 1B). Moreover, recombinant NleC cleaved the N-terminally FLAG-tagged p65 protein, with the tag allowing for better resolution of the small N-terminal fragment (Fig. 1C). Of note, although recombinant NleC was able to cleave the ectopically expressed FLAG-tagged p50 protein, the product size marked by the N- and C-terminal FLAG tag indicates that cleavage occurs at the C-terminus of p50 rather than the conserved cysteine 62/glutamic acid 63 in the N-terminus (S4 Fig), which corresponds to C38/E39 in p65. In addition, recombinant NleC failed to cleave ectopically expressed RPS3, which is a non-Rel subunit of NF-κB that confers the promoter selectivity and transcriptional specificity [34], in the recombinant protease cleavage assay (S5 Fig). Therefore, NleC appears to selectively cleave p65, but not other NF-κB subunits.

### The N-terminal 20 amino acids of p65 are essential for NleC-mediated cleavage

**Fig 2:**
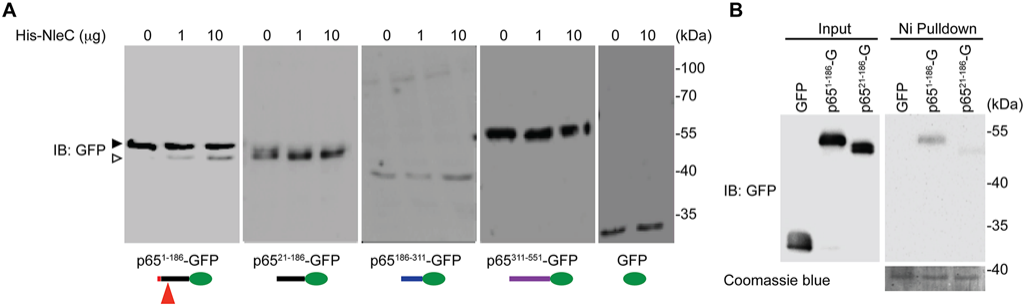
The N-terminal 20 amino acids of p65 are required for NleC to bind and cleave p65. **A**. Various truncations of C-terminal GFP-tagged p65, as illustrated at the bottom, were transfected into HEK293T cells. Whole cell lysates were subjected to the His-NleC cleavage assay, and immunoblotted with anti-GFP antibody for NleC-cleaved fragments. The GFP-tagged p65^1–186^ protein and cleaved fragment were labeled by filled and open triangles, respectively, and the NleC cleavage sites in p65 were indicated below. **B**. Whole cell lysates derived from HEK293T cells expressing GFP or the indicated GFP-tagged p65 proteins were incubated with the catalytic mutant His-NleC (H117Y) at 4°C. Nickel beads were added to pull-down His-NleC and associated proteins. Samples were separated by SDS/PAGE, followed by immunoblot for indicated proteins.

Notably the first 20 amino acids of p65 are unique to this protein and not conserved among the Rel proteins (Fig. 1A and S1 Fig), providing a clue to why NleC cleaves p65 specifically, despite the fact that the C38/E39 cleavage site is shared by all other Rel subunits. Protease-substrate interaction-induced conformational changes are known to play an important role in the optimal cleavage of substrates by proteases [46]. We therefore hypothesized that the unique N-terminal sequence (a.a. 1–20) could be critical for the interaction between p65 and NleC, and be a prerequisite for NleC-mediated p65 cleavage. Using a library of C-terminal GFP-tagged p65 truncation constructs [34], we mapped the necessary region(s) of p65 for the NleC-conferred cleavage. Interestingly, recombinant NleC cleaved the p65^1–186^ truncation as expected, whereas the p65^21–186^ truncation harboring the C38/E39 cleavage site but missing the first 20 amino acids was not cleaved by NleC, even in the presence of an overwhelming amount (10 μg) of the recombinant protease (Fig. 2A). To confirm this, we moved the GFP tag to the N-terminus of p65^21–186^ truncation to increase resolution of the cleavage product and we were still unable to detect cleavage (S6 Fig). These data suggest that the N-terminal 20 residues and C38/E39 cleavage site are both required for NleC-mediated p65 cleavage. In support of this notion, the full-length p65 containing both elements was cleaved by NleC, whereas no cleavage was detected in the p65^186–311^ and p65^311–551^ truncations as well as GFP vehicle control that do not contain either element (Fig. 2A, 3A-3B and S6 Fig). Together these results demonstrate that the first 20 amino acids are essential for NleC-mediated cleavage to occur, thus providing a rational for the specificity of NleC for p65 rather than other Rel homology proteins, despite a shared Rel homology domain sequence.

**Fig 3:**
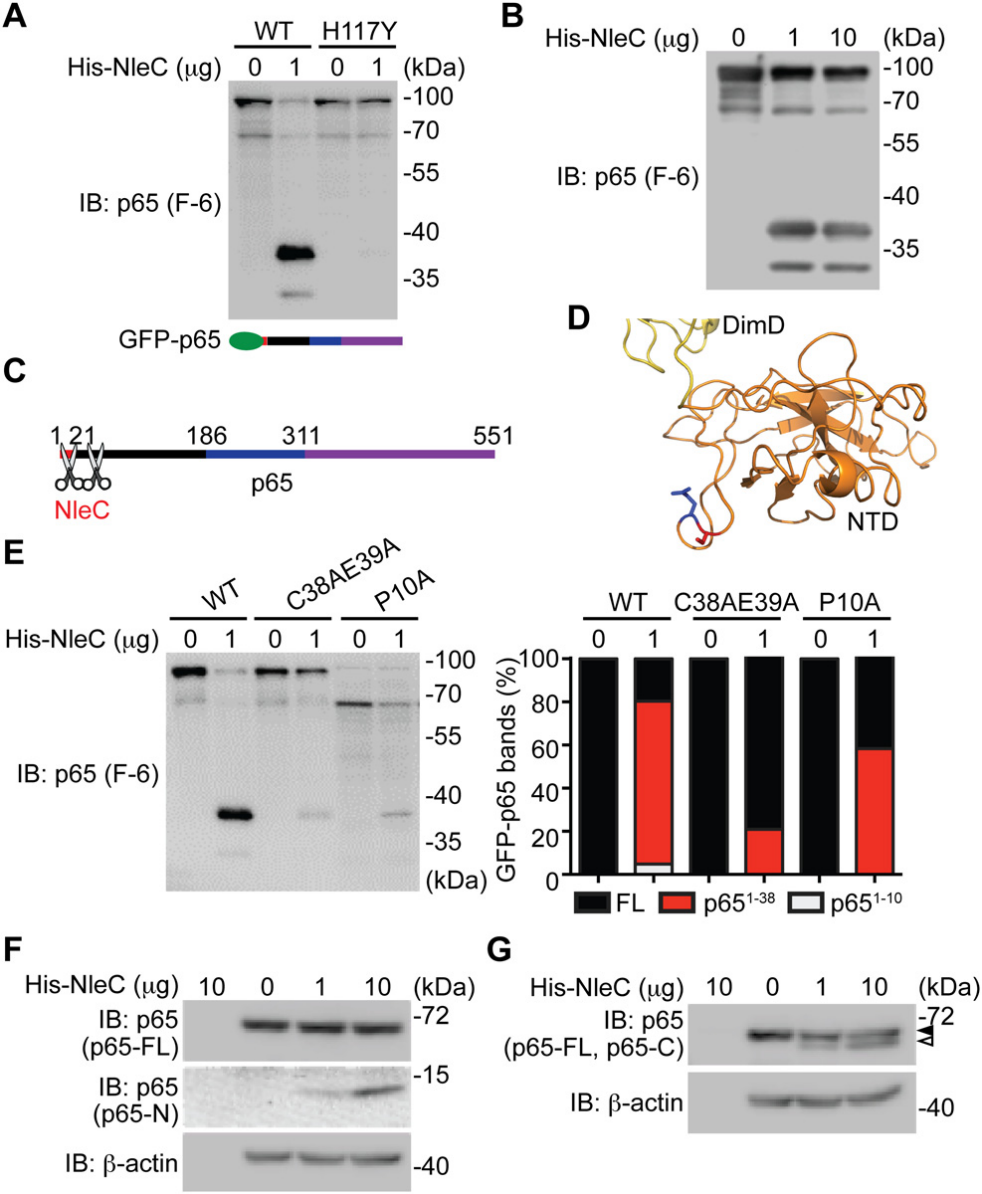
The dominant NleC cleave site on p65 is Cys38/Glu39. **A-B**. Whole cell lysates derived from HEK293T cells expressing N-terminal GFP-tagged p65 were subjected to the cleavage assay using wild-type His-NleC or the catalytic mutant His-NleC (H117Y), and immunoblotted with p65 N-terminus specific antibody for NleC-cleaved p65 fragments. **C**. Schematic of proposed NleC cleavage sites on p65. **D**. The structure of N-terminus of p65, with dimerization domain (DimD), N-terminal domain (NTD), Cys38, and Glu39 highlighted in yellow, orange, red, and blue, respectively. Image was created from PDB file 1VKX [47] using the Pymol software. **E**. Whole cell lysates derived from HEK293T cells expressing wild-type or mutant GFP-tagged p65 as indicated were subjected to the NleC cleavage assay and immunoblotted with p65 N-terminus specific antibody for NleC-cleaved p65 fragments. The percentage of GFP-tagged full-length p65, p65^1–38^ fragment, and p65^1–10^ fragment among the total GFP-tagged p65 proteins under each condition was quantified by densitometry. **F-G**. Whole cell lysates derived from HEK293T cells were subjected to the His-NleC cleavage assays, and immunoblotted with p65 N-terminus (F) and C-terminus (G) specific antibodies for NleC-cleaved p65 fragments.

### The N-terminal 20 amino acids of p65 are required for NleC recognition

It has been widely acknowledged that the recognition of a substrate by a protease is critical for subsequent conformational changes to form a stabilized tetrahedral intermediate and to initiate optimal cleavage [46]. To examine whether the N-terminal 1–20 residues of p65 are required for NleC to recognize and bind p65, we incubated the whole cell lysates from HEK293T cells expressing full-length or truncated p65, with catalytically inactive NleC (H117Y) mutant or wild-type NleC at 4°C and conducted pull-down assays using nickel beads. NleC associated with full-length p65, but did not interact with p65^311–551^ (S7 Fig), in line with the evidence that NleC cleaves full-length p65 rather than the truncated p65^311–551^ (Fig. 2A). Moreover, a significant interaction between NleC (H117Y) and p65^1–186^ was detected, whereas there was little, if any, interaction between NleC (H117Y) and p65^21–186^ (Fig. 2B and S7 Fig.), suggesting that the N-terminal 20 amino acids could be the key targeting sequence for NleC to specifically recognize and bind to p65.

### The major NleC cleavage site on p65 is between cysteine 38 and glutamic acid 39

Previous studies showed that C38/E39 is the NleC cleavage site in p65 [41,44,45], whereas P10/A11 was also reported as an NleC cleavage site [16]. We therefore conducted recombinant NleC cleavage assays using lysates containing p65 N-terminally tagged with GFP, which allowed us to further examine the cleavage location(s) on the N-terminus of p65. In agreement with our previous results (Fig. 2A), recombinant NleC cleaved p65 at the N-terminus generating two fragments that were N-terminally tagged with GFP; the cleaved products, which migrate at 37 kDa and 30 kDa, respectively, were verified using an antibody that specifically recognizes the N-terminus of p65 (Fig. 3A-3B). Of note, substantially more GFP-tagged p65^1–38^ fragment was detected, indicating that NleC chiefly cleaves p65 at C38/E39. Therefore our results, in line with previous reports [16,41,44,45], demonstrate that the N-terminus of p65 harbors two NleC cleavage sites, P10/A11 and C38/E39, of which C38/E39 is the primary cleavage site whereas P10/A11 appears to be the secondary one (Fig. 3C). In support of this notion, the resolved p65 crystal structure [47] reveals that the C38/E39 residues are located in a surface loop of the protein, which facilitates protease access (Fig. 3D). Moreover, the NleC-generated p65^1–38^ fragment is substantially reduced by an alanine substitution to C38/E39 (Fig. 3E). However, the NleC-cleaved p65^1–10^ fragment was also abolished by the C38A/E39A mutation. In contrast, the NleC-generated p65^1–38^ fragment is less profoundly impacted by the alanine substitution to P10, despite a complete inhibition of the NleC mediated cleavage of p65 at P10/A11 (Fig. 3E). These results therefore strengthen our conclusion that C38/E39 is the major NleC cleavage site within p65. Moreover, lysates from normal HEK293T cells were subjected to the NleC cleavage assays to examine how recombinant NleC cleaves endogenous p65. As detected by an antibody specific for the p65 N-terminus, a p65 fragment migrating as approximately 10 kDa on SDS/PAGE gels was generated by NleC in a dose dependent fashion (Fig. 3F). Because cleavage of p65 at P10/A11 would result in a fragment approximately 1 kDa in size that would likely be degraded, we therefore determined the cleaved p65 fragment is most likely the product of cleavage at C38/E39. Likewise, we observed a larger C-terminal fragment, migrating around 60 kDa on SDS/PAGE, using an antibody specific for the C-terminus of p65 (Fig. 3G). Together, our results suggest that C38/E39 is the major NleC cleavage site on p65, which generates a detectable p65^1–38^ fragment.

### NleC cleaves p65 and generates the p65^1–38^ fragment during *C*. *rodentium* and EPEC infections

**Fig 4:**
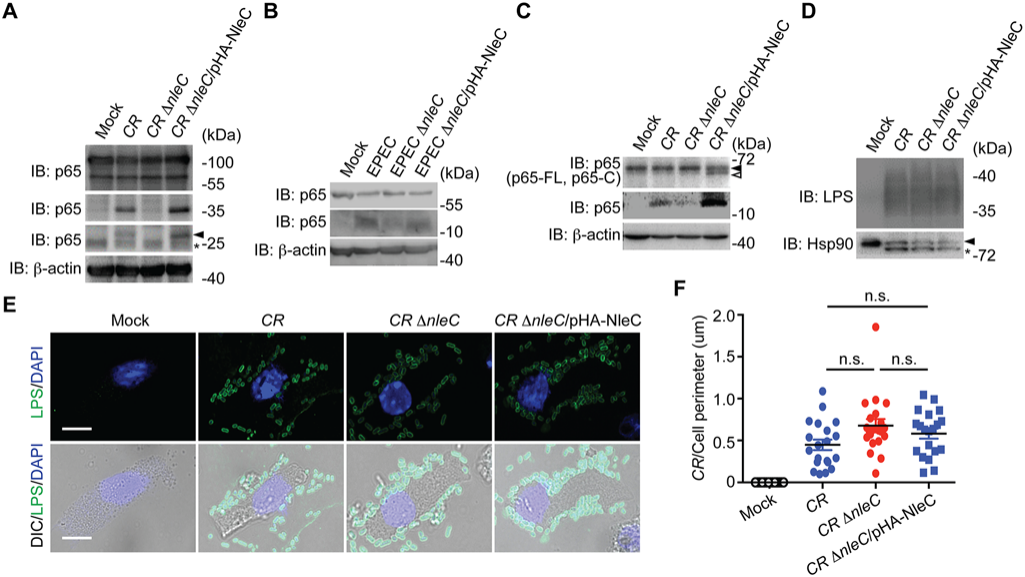
NleC cleaves p65 during *C*. *rodentium* and EPEC infections. **A**. HEK293T cells expressing N-terminal GFP-tagged p65 were mock infected or infected with indicated *C*. *rodentium* strains at 100 MOI for 3 h. Whole cell lysates were derived, separated by SDS/PAGE, and immunoblotted for indicated proteins. The GFP-tagged p65^1–10^ cleavage products are indicated by an arrow, and an asterisk labels nonspecific bands. **B**. Caco-2 cells were mock infected or infected with indicated EPEC strains at 100 MOI for 1 h. Whole cell lysates were derived, separated by SDS/PAGE, and immunoblotted for indicated proteins. **C**. Mouse colon epithelial cells (CECs) were mock infected or infected in suspension with indicated *C*. *rodentium* strains at 100 MOI for 3 h. Whole cell lysates were derived, separated by SDS/PAGE, and immunoblotted for indicated proteins. The full-length p65 and cleaved p65 C-terminal fragment are indicated by filled and open triangles, respectively. **D**. Mouse CECs infected as in C were separated from free *C*. *rodentium* by Percoll gradient centrifugation. Whole cell lysates from CECs and attached *C*. *rodentium* were derived, separated by SDS/PAGE, and immunoblotted for indicated proteins. **E**. Representative immunofluorescence micrographs of mouse CECs infected as in C that were centrifuged onto cover slips and stained for *C*. *rodentium* LPS, with nuclei counterstained by DAPI. Scale bars, 10 μm. **F**. The numbers of *C*. *rodentium* attached to mouse CECs as in E (from 6 random fields) were quantified and normalized to the perimeter of individual CEC.

To further examine the pathophysiological relevance of NleC-mediated p65 cleavage, we employed the EPEC and *C*. *rodentium* infection models. After 3-hour infection of wild-type *C*. *rodentium*, we detected the p65^1–10^ and p65^1–38^ fragments in HEK293T cells expressing GFP-tagged p65 (Fig. 4A). The p65^1–10^ and p65^1–38^ cleavage was abolished in the cells infected with a *C*. *rodentium* mutant strain lacking NleC (*ΔnleC*) [20], compared to wild-type *C*. *rodentium* (Fig. 4A). Moreover, the attenuated p65 cleavage was robustly restored in the cells infected with a *C*. *rodentium* mutant strain that lacks NleC but was complemented with a HA-NleC plasmid (*ΔnleC*/pHA-NleC) [20] (Fig. 4A). Notably, we also observed the NleC-cleaved endogenous p65^1–38^ fragment in Caco-2 cells, a human colon cancer cell line, and isolated mouse primary colon epithelial cells (CECs) infected by wild-type EPEC and *C*. *rodentium*, respectively (Fig. 4B and 4C). Strikingly, the p65^1–38^ fragments were greatly diminished in cells infected with *ΔnleC* mutant bacteria; whereas the p65 cleavage was rescued in cells infected with complemented *ΔnleC*/pHA-NleC strains (Fig. 4B and 4C), suggesting that NleC cleaves p65 thus generating the p65^1–38^ fragment during EPEC and *C*. *rodentium* infections in cell culture. In contrast to p65, the cleavage of p50 in Caco-2 cells and mouse CECs infected by EPEC and *C*. *rodentium*, respectively, was not detectable (S8 Fig), further supporting that NleC specifically cleaves p65 among NF-κB subunits. To examine the possibility that the abolished p65 cleavage in CECs infected with *ΔnleC C*. *rodentium* could be due to defective attachment to host cells, in comparison to wild-type and complemented strains, we measured the attachment of variant *C*. *rodentium* strains to CECs during infection. As shown in Fig. 4E, wild-type, *ΔnleC*, and *ΔnleC*/pHA-NleC *C*. *rodentium* attached to CECs in a similar pattern. Moreover, as assayed by enumeration of CEC-attached bacteria and immunoblot for marker proteins in mouse CECs (heat shock protein, Hsp90) and *C*. *rodentium* (lipopolysaccharides, LPS), the amount of *ΔnleC* and *ΔnleC*/pHA-NleC *C*. *rodentium* that attaches to CECs during infection was equal if not higher than the wild-type bacteria (Fig. 4D and 4F). These results rule out the possibility that NleC deletion affects the interaction between *C*. *rodentium* and CECs, and suggest that NleC executes the p65 cleavage during EPEC and *C*. *rodentium* infections, generating a small amount of p65^1–38^ fragment and leaving most p65 intact.

### NleC affects host immune responses in mice infected by *C*. *rodentium*

**Fig 5:**
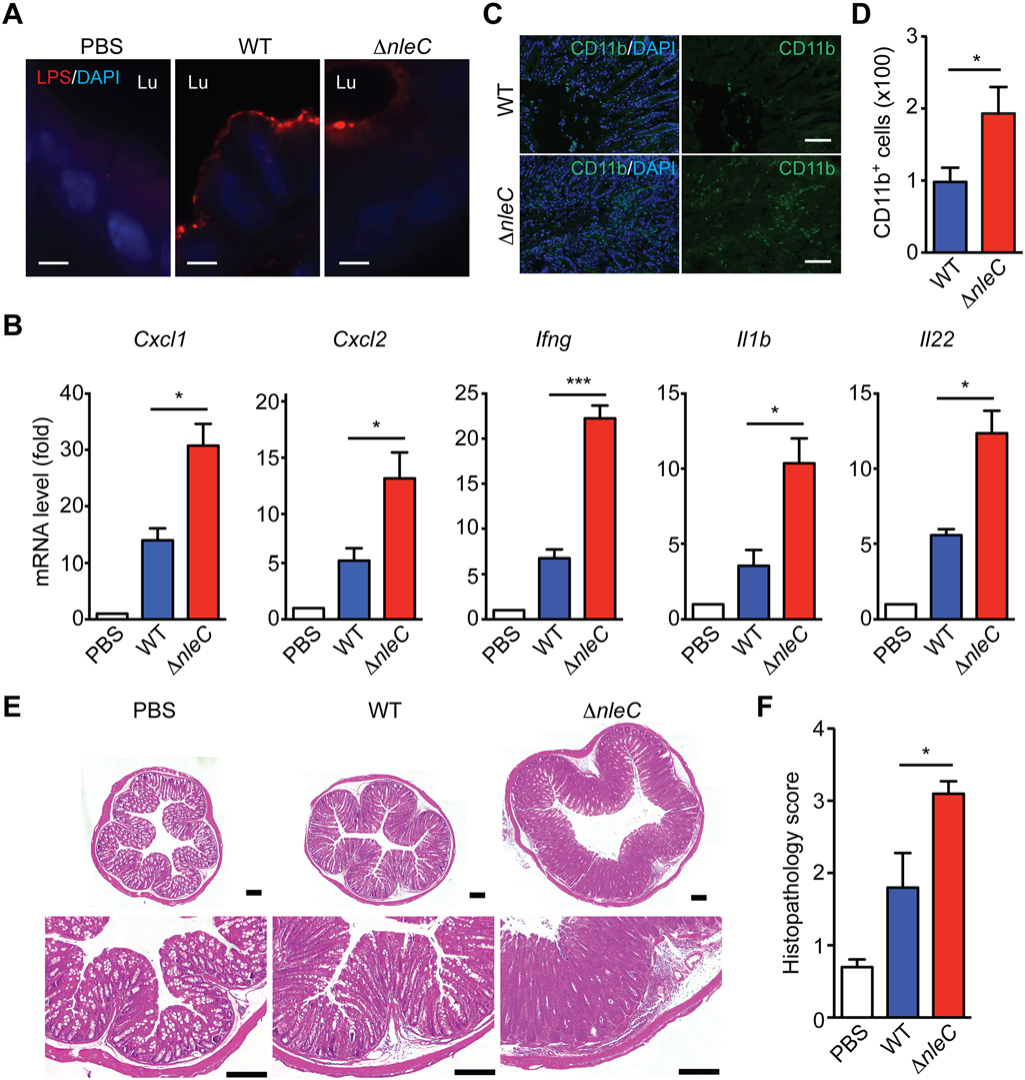
NleC suppresses inflammatory responses in *C*. *rodentium*-infected mice. **A**. Representative immunofluorescence micrographs of *C*. *rodentium* LPS, with nuclei counterstained by DAPI, in colon sections derived from C57BL/6 mice 8 days post inoculation with the indicated strains of *C*. *rodentium*. Scale bars, 10 μm. Lu indicates the colon luminal space. **B**. Quantitative PCR (qPCR) was used to determine mRNA levels of indicated cytokine/chemokine genes relative to *Actb* (β-actin) in the colons collected from C57BL/6 mice 14 days post inoculation with wild-type or *ΔnleC* mutant strain of *C*. *rodentium*. **C-D**. Immunofluorescence micrographs of CD11b^+^ inflammatory immune cells in the colons collected from C57BL/6 mice as in B, with nuclei counterstained by DAPI. Scale bars, 10 μm. The colon-infiltrated inflammatory immune cells (from 5 random fields) with CD11b staining were quantified (D). **E**. Hematoxylin and eosin (H&E) staining of colons collected from C57BL/6 mice as in D. Scale bars, 200 μm. **F**. The histopathology scores of colon sections derived from mice infected as indicated as in D. Shown are mean ± s.e.m of 10 random fields from two independent experiments.

Infection of *C*. *rodentium* in mice is known to cause colonic epithelial damage by acute inflammatory responses [48], therefore we examined the impact of NleC on colonic inflammatory response in mice inoculated with variant strains of *C*. *rodentium*. As we reported previously [20], the colonization of wild-type and *ΔnleC* mutant *C*. *rodentium* in the colon of infected mice was comparable at day 8 and day 10 (Fig. 5A and S9 Fig, respectively). In line with previous reports that NleC-mediated p65 cleavage plays a critical role in dampening the NF-κB signaling pathway and suppressing proinflammatory gene expression in EPEC-infected cells [16,30], we observed robust transcription of known NF-κB target genes *Cxcl1* and *Cxcl2* in the colon tissues removed from mice infected with *ΔnleC C*. *rodentium*, compared to wild-type bacterium at day 14 (Fig. 5B). We also detected significantly elevated levels of additional NF-κB target genes *Ifng*, *Il1b*, and *Il22*, thus highlighting the key function of NleC-mediated p65 cleavage in interfering with host NF-κB gene transcription (Fig. 5B). Consistent with the colonic expression of proinflammatory cytokine/chemokine genes, the amount of infiltrated CD11b^+^ myeloid cells in the colon was increased by two fold in mice orally inoculated with *ΔnleC C*. *rodentium*, compared to that from mice infected with wild-type bacterium (Fig. 5C-5D). As expected, wild-type *C*. *rodentium*-infected mice developed severe clinical symptoms characterized by crypt elongation, thickening of the mucosal surface, and goblet cell depletion in histological staining, in comparison to phosphate-buffered saline (PBS) inoculated animals (Fig. 5E-5F). By contrast, infection with *ΔnleC C*. *rodentium* induced more severe damage to colon epithelia and general enlargement of the colonic tissue (Fig. 5E-5F). Our results therefore demonstrate that the p65 cleavage by NleC during *C*. *rodentium* infection has a dramatic effect on NF-κB gene transcription and inflammatory response in the infected animals.

### The NleC-cleaved p65^1–38^ interacts with RPS3

**Fig 6:**
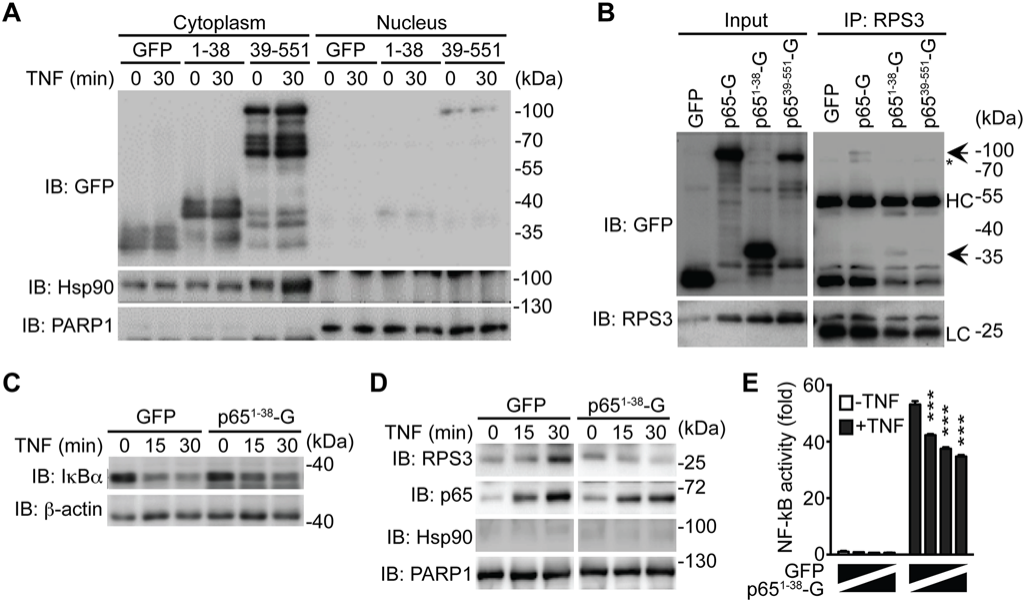
The p65^1–38^ fragment interferes with the RPS3-p65 interaction in the cytoplasm and selectively attenuates the nuclear translocation of RPS3. **A**. HEK293T cells were transfected with C-terminal GFP-tagged p65^1–38^, p65^39–551^, or GFP control plasmids. 28 h later, the cytosolic and nuclear fractions were derived and immunoblotted (IB) for indicated proteins. Hsp90 and PARP1 served as loading controls and cytosolic and nuclear markers, respectively. **B**. HEK293T cells were transfected with indicated GFP-tagged p65 or GFP control plasmids. 28 h later, whole cell lysates (Input) were derived and directly IB, or after immunoprecipitation (IP) with RPS3 antibody, for indicated proteins. Non-specific bands and immunoprecipitated GFP-p65 proteins are indicated by asterisks and arrows, respectively. HC, heavy chain; LC, light chain. **C**. HEK293T cells expressing GFP-tagged p65^1–38^ or GFP alone were stimulated with 50 ng ml^-1^ of TNF for indicated periods. Whole cell lysates were derived and IB for IκBα, with β-actin as a loading control. **D**. HEK293T cells were transfected and stimulated as in C, and nuclear fractions were derived and IB for indicated proteins. Hsp90 and PARP1 served as loading controls and cytosolic and nuclear markers, respectively. **E**. HEK293T cells were transfected with increasing amounts of GFP-p65^1–38^, compensated with GFP control, together with 5 × κB-Luc reporter and pTKRL plasmids. After 28 h, the cells were stimulated in the presence or absence of TNF (50 ng ml^-1^) and analyzed for luciferase activity.

It is noteworthy that NleC cleaved only a small percentage of p65 during *C*. *rodentium* infection (Fig. 4A-4C) and even in the presence of an overwhelming amount (10 μg) of recombinant protease (Fig. 1B, 2A, 3A-3B, 3F-3G). In particular, the dramatic effect of NleC on the proinflammatory cytokine production and NF-κB activity was proposed to be mediated by the cleavage of p65 by NleC [16,20,29,30,41,42]; however, the large amount of full-length p65 resistant to NleC cleavage makes it difficult to explain the remarkable impact of NleC on dampening host NF-κB signaling and inflammatory response. Of note, RPS3, a non-Rel subunit of NF-κB, was revealed to confer the promoter selectivity and transcriptional specificity of NF-κB [34,49], in particular the RPS3/NF-κB-mediated transcription of a subset of proinflammatory genes is critical for host defense against A/E pathogens [17,18,19]. Moreover, our previous studies showed that interrupting the subcellular localization and function of RPS3 by small interfering RNA (siRNA) [34], bacterial effectors [17,18,19], and ectopic expression of an N-terminal truncated p65^21–186^ fragment [40], are able to selectively block NF-κB target gene transcription, without affecting the nuclear translocation of p65. We therefore hypothesized that the NleC-cleaved p65^1–38^ product would execute a similar function as the p65^21–186^ fragment [40], which interferes with RPS3 signaling. To test this hypothesis, we examined the ability of GFP-tagged p65^1–38^ and p65^39–551^ truncated proteins to alter NF-κB signaling in cultured cells. As demonstrated by subcellular fractionation, both p65^1–38^ and p65^39–551^ truncated proteins were primarily located in the cytoplasm of transfected HEK293T cells, even following 30-min TNF treatment (Fig. 6A). This result is in agreement with previous reports that NleC inactivates p65 in the cytoplasm [16,41], and suggests that the NleC-cleaved products would mainly interfere with cytoplasmic NF-κB signaling in host cells. We further examined the interaction between RPS3 and the GFP-tagged p65^1–38^ and p65^39–551^ fragments by immunoprecipitation. The RPS3-p65^1–38^ interaction was comparable to, if not even stronger than, that of RPS3 and full-length p65, whereas the association between RPS3 and the p65^39–551^ fragment was barely detectable (Fig. 6B). Moreover, the p65^1–38^ truncated protein was substantially enriched in the GST-RPS3 pulldown sample, compared to the GST vehicle control (S10 Fig), which independently verifies the interaction between p65^1–38^ and RPS3. Our results therefore suggest that the NleC-cleaved p65^1–38^ product, rather than the p65^39–551^ fragment, of p65 is able to interact with the NF-κB non-Rel subunit RPS3.

### The p65^1–38^ product selectively attenuates the nuclear translocation of RPS3

**Fig 7:**
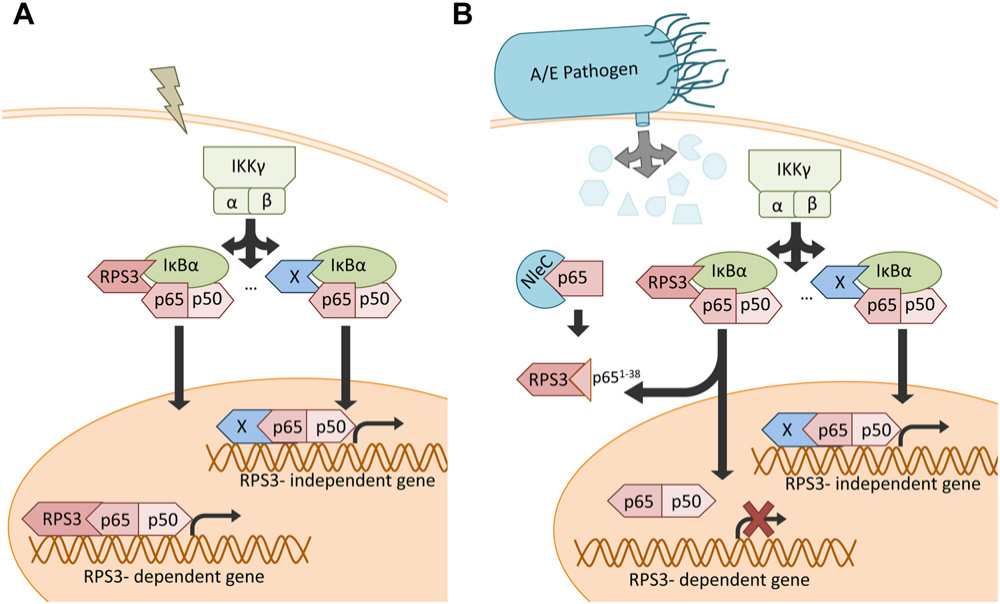
Schematic model of selective inhibition of NF-κB gene expression by the NleC-cleaved p65^1–38^ fragment. **A**. Under normal conditions, NF-κB stimuli activate both RPS3-dependent and-independent NF-κB signaling pathways. **B**. When injected into host cells via the T3SS during A/E pathogen infections, NleC cleaves a small percentage of p65, generating the p65^1–38^ fragment that interferes with the RPS3-p65 interaction and sequestrates RPS3 in the cytoplasm. This leads to selective inhibition of the RPS3-dependent NF-κB gene transcription, without affecting the RPS3-independent gene transactivation.

Stimuli-triggered translocation from the cytoplasm to the nucleus is a prerequisite for RPS3 to facilitate NF-κB binding and transactivation of specific target genes [33,49]. We recently showed that ectopic expression of p65^21–186^ truncated protein competed RPS3 off endogenous full-length p65, thereby interfering with the nuclear translocation of RPS3 during the NF-κB response [40]. Ectopic expression of the p65^1–38^ fragment, compared to the GFP control, did not alter TNF-stimulated IκBα degradation (Fig. 6C) and p65 nuclear translocation (Fig. 6D). By contrast, overexpression of p65^1–38^ fragment remarkably attenuated TNF-triggered nuclear translocation of RPS3, which was induced normally in the GFP-expressing cells (Fig. 6D). To further assess the impact of p65^1–38^ fragment on NF-κB activation, we examined the expression of an Ig-κB site-driven luciferase reporter gene, which was previously shown to be RPS3-dependent [18,34,40], in HEK293T cells expressing the p65^1–38^ fragment. Indeed, in comparison to the GFP vehicle control, ectopic expression of the p65^1–38^ fragment attenuated NF-κB reporter luciferase expression in a dose dependent manner (Fig. 6E). These results suggest that the p65^1–38^ fragment is capable of interfering with the endogenous p65/RPS3 interaction and selectively attenuating the nuclear translocation of RPS3 rather than affecting other branches of NF-κB signaling (Fig. 7), which provides a novel mechanism for A/E pathogens to specifically modulate host NF-κB-mediated gene transcription and inflammatory responses.

## Discussion

The NF-κB signaling pathway orchestrates both innate and adaptive immune responses in host cells, thereby executing an important function in host defense to a variety of pathogens [21]. That said, the mechanisms controlling promoter selectivity and transcriptional specificity of NF-κB genes in acute pathogen-host interactions remain obscure. Beyond the well-characterized NF-κB Rel subunits, we recently identified two non-Rel subunits, *i*.*e*. RPS3 and Sam68, in the NF-κB DNA binding complexes that confer distinct transcriptional specificity of NF-κB [33,49]. The RPS3- and Sam68-conferred NF-κB activation model suggests that the Rel subunits are required, but not the sole determinants, for the activation of NF-κB target genes. In contrast, the synergistic interactions between Rel and non-Rel subunits at the promoters are critical for the transactivation of certain NF-κB target genes [33,49]. An increasing number of studies [17,18,34,37,38,39] suggest that the RPS3/NF-κB signaling pathway is vital in the host proinflammatory transcription and immune responses against infection by A/E pathogens [17,18]. Specifically, the T3SS effector NleH1 from EPEC, EHEC, and *C*. *rodentium* attenuates the nuclear translocation of RPS3, but not p65, during NF-κB activation by inhibiting the IKKβ-mediated Ser209 phosphorylation of RPS3 [17,18]. This leads to reduced transcription of select, but not all, NF-κB target genes, most of which are RPS3/NF-κB-dependent proinflammatory cytokine genes [17,18]. In this work, we show that the T3SS effector protease NleC predominantly cleaves p65 at C38/E39. The NleC cleavage-generated p65^1–38^ fragment interferes with the RPS3/p65 interaction in the cytoplasm, resulting in the attenuated nuclear translocation of RPS3, without affecting p65 nuclear translocation. Similar to what we have shown previously [17,18,34,40], cytoplasmic sequestering of RPS3 prevents the transactivation of the RPS3/p65-dependent proinflammatory cytokine genes and the infiltration of inflammatory immune cells to infected tissue. Of note, NleC cleaves a small percentage of p65 in *C*. *rodentium*-infected mouse CECs. In contrast, NleC cleavage has a substantial effect on proinflammatory cytokine gene transcription, as evidenced by infections using the EPEC and *C*. *rodentium* genetic mutants with disruptions in the NleC gene, compared to wild-type strains. We are aware that NleC, as a metalloprotease, cleaves other host proteins beyond p65, such as p300 [42], p38 [20], and others, which could also result in the observed striking effects of NleC on colonic cytokine gene expression and inflammatory responses in infected mice. However, our proposed “amplifying” mechanism through the RPS3-conferred NF-κB specific transcription provides a novel explanation for the disconnect regarding the extent of NleC-mediated NF-κB cleavage and the impact of NF-κB transactivation by such cleavage. In conjunction with our previous studies showing that NleH1 attenuates RPS3 Ser209 phosphorylation [17,18], our results highlight the critical role of the RPS3/NF-κB signaling pathway in the host immune response to A/E pathogen infections. Although NleC and NleH1 are both secreted by the A/E pathogen T3SS, the kinetics and amount of NleC and NleH1 within host cells during infection remains largely unknown. It has been well documented that certain successful pathogens have acquired sophisticated mechanisms to directly interfere with host NF-κB signaling through regulating or mimicking host proteins to their own advantage, and these co-opted strategies can operate at multiple levels of the sequential process of NF-κB signaling [11,12,13,14,15,16,17,18,29,30]. We therefore speculate that A/E pathogens utilize NleC and NleH1 to simultaneously (or sequentially) interfere with the RPS3/NF-κB signaling pathway through distinct strategies, therefore ensuring with this redundancy that the NF-κB signaling and inflammatory responses are dampened in host cells. Notably, the RPS3-p65 branch of NF-κB signaling has been associated with transcription of anti-apoptotic genes [39] and inflammatory genes [17,18] therefore selectively targeting this arm could prove advantageous to the pathogen. Given that the spatial and temporal coordination of effectors injected into host cells remains unclear [50,51], it is intriguing to consider that manipulating inflammatory gene transcription would be valuable early during infection and ensuring apoptosis would be an escape strategy allowing the pathogen to spread to the next host. While it has been documented that a relatively low amount of NleC is delivered into host cells during infection in cultured cells [9], the timing, longevity, and activity of the effectors within the host cell during an *in vivo* animal infection remains elusive. Greater resolution of the timing and amount of NleC’s introduction into the host cell and interaction with p65 is still needed.

The most abundant species among the NF-κB complexes in cells consists of p65, p50, and other proteins [34]. Moreover, p65 possesses a transactivation domain (TAD) that is essential to recruit general transcriptional machinery to transcribe target genes, whereas p50 lacks the TAD domain thereby normally suppressing gene expression [33]. These features make p65 unique within the NF-κB Rel subunits and a likely target for pathogen effectors. Our results show that NleC cleaves p65 more efficiently than other Rel family proteins and non-Rel subunits, RPS3 and Sam68. In further support of this notion, the N-terminal 20 residues of p65, which are not conserved among other Rel family proteins, play a critical role in the recognition and cleavage of p65 by NleC during A/E pathogen infection. Therefore it is not surprising that p65, the most important NF-κB Rel subunit, is a major target for a wealth of pathogens, allowing them to interfere with the NF-κB signaling pathway in host cells [16,20,29,30,41]. Indeed, previous studies showed that p65 was cleaved by a myriad of pathogen encoded proteases [52,53,54,55,56,57], although the direct consequence of cleaving p65 on NF-κB signaling has not been extensively studied. For instance, *Chlamydia trachomatis*, a gram-negative bacterium that causes urethritis, cervicitis, and other diseases, encodes a PDZ containing tail-specific protease [58]. The enzyme was proposed to suppress host NF-κB activity by cleaving p65 and generating two p65 fragments (approximately 40 kDa and 25 KDa, respectively) [59]. Moreover, the A-B toxin metalloprotease encoded by the fish pathogen *Photobacterium damselae* piscicida was recently shown to cleave p65 at C38/E39, similar to NleC [52]. It would be interesting to examine if p65 fragment interference is a previously unrecognized but possibly widespread mechanism of virulence for abrogating host NF-κB signaling, especially when pathogen encoded proteases are unable to cleave the majority or entire cellular source of p65. By selectively blocking NF-κB “specifiers”, as we demonstrated here for RPS3, pathogens could more acutely manipulate the host environment by regulating the timing and abundance of injected effectors, allowing collections of genes to be turned on and off to their advantage.

## Materials and Methods

### Ethics statement

All animal experiments were performed according to protocol number MO13-H349, approved by the Johns Hopkins University’s Animal Care and Use Committee and in direct accordance with the NIH guidelines for housing and care of laboratory animals.

### Cell line, antibodies, and plasmids

HEK293T and Caco-2 cells (ATCC, Manassas, VA) were cultured in DMEM medium containing 10% fetal calf serum, 2 M glutamine, 100 U ml^-1^ penicillin, and 100 U ml^-1^ streptomycin. Antibodies used were: p65 (C-terminus, C-20, sc-372), p65 (N-terminus, F-6, sc-8008x), p50 (NLS, sc-114), c-Rel (C, sc-71) from Santa Cruz Biotechnology (Dallas, TX); β-actin (AC-15, A5441) and FLAG (M2, F1804) from Sigma-Aldrich (St. Louis, MO); PARP-1 (46D11, 9532) from Cell Signaling Technology (Danvers, MA); GFP (7.1 and 13.1, 11814460001) from Roche Applied Science (Indianapolis, IN); CD11b (M1/70, 101202) from BioLegend (San Diego, CA); Hsp90 (610418) from BD Biosciences (San Jose, CA); *E*. *coli* O152 LPS (81449) from Statens Serum Institut (Copenhagen, Denmark); RPS3 as previously described [34]. Tumor necrosis factor (TNF) was purchased from R&D System (Minneapolis, MN). The plasmids His-NleC [30], FLAG-p50, p50-FLAG [60], GFP-tagged full-length p65, p65^1–186^, p65^21–186^, p65^1–311^, p65^186–311^, and p65^311–551^ [34] were previously described. The GFP-p65^1–38^ and GFP-p65^39–551^ were generated by inserting the appropriate fragments into the pEGFP-N1 vector (Clontech Laboratories, Mountain View, CA) using the InFusion Cloning System (Clontech Laboratories). The His-NleC (H117Y) and GFP-tagged p65 (C38A/E39A) and p65 (P10A) mutants were generated by site-directed mutagenesis using the Quick Change Kit (Stratagene, La Jolla, CA) with appropriate primers. All the plasmids were verified by DNA sequencing.

### Transient transfection

DNA constructs were transfected into HEK293T cells using the TurboFect *in vitro* transfection reagent (Thermo Scientific, Waltham, MA) according to the manufacturer’s instructions, as described previously [40].

### NleC protease digestion assays

Overnight cultures of BL21 (pET-NleC) were induced with1 mM IPTG and His-NleC proteins were purified by nickel affinity chromatography. The NleC protease digestion was conducted as previously described [30]. Briefly, cells were collected and lysed on ice with 0.4 ml of lysis buffer (50 mM Tris-HCl [pH 8.0], 150 mM NaCl, 1% NP-40 and 0.5% sodium deoxycholate, 1 × complete protease inhibitor cocktail [Roche Applied Science]) for 30 min. After centrifuge at 10,000 × *g* at 4°C for 10 min, 200 μl of supernatant was removed to a separate tube and incubated with indicated amount of His-NleC protein at 37°C for 3 h.

### Nickel bead and GST pull-down assays

For the interactions between NleC and indicated proteins, the 200 μl of supernatant mixed with 1 μg of His-NleC protein were subjected to pull-down assays by adding 30 μl of Nickel beads (Qiagen, Germantown, MD), and rotating for 30 min at 4°C. The GST pulldown assays were conducted as previously described [35]. The pull-down proteins were washed at least four times with cold lysis buffer followed by separation with SDS-PAGE.

### Isolation of primary colon epithelial cells

Colon epithelial cells (CECs) were isolated from C57BL/6J mice as previously described [61]. Briefly, after euthanizing mice, the entire colon was removed under aseptic conditions and washed twice with ice-cold PBS. After dividing the colon into 2–3 mm long fragments and transferring them into chelating buffer (27 mM trisodium cirtcrate, 5 mM Na_2_HPO_4_, 96 mM NaCl, 8 mM KH_2_PO_4_, 1.5 mM KCl, 0.5 mM DTT, 55 mM D-sorbitol, 44 mM sucrose, 6 mM EDTA, 5 mM EGTA [pH 7.3]) for 45 min at 4°C, CECs were then dislodged by repeated vigorous shaking. Tissue debris was removed by a 70-μm cell strainer (Fisher Scientific, Suwanee, GA) and CECs were harvested by centrifugation at 4°C. The viability of CECs was confirmed by trypan blue staining and isolated CECs were cultured at 37°C for 1 h for recovery, followed by infection.

### *Citrobacter rodentium* and EPEC growth conditions and infection in cultured cells

Wild-type *C*. *rodentium* (DBS 100) and EPEC (E2348/69), as well as the NleC deletion mutant (*ΔnleC*) and the HA-NleC complemented (*ΔnleC*/pHA-NleC) strains [20] were grown from single colonies on Luria-Bertani (LB) plates in LB broth at 37°C overnight with shaking. Infection of EPEC in Caco-2 cells was performed as previously described [20]. Prior to infection experiments, *C*. *rodentium* was washed with ice-cold PBS and resuspended in pre-warmed corresponding media. Bacteria concentration was measured by absorbance at optical density 600, followed by a serial dilution and seeding on a MacConkey agar plate (VWR, Radnor, PA) to confirm the administered colony-forming units (CFU). The HEK293T cells and isolated CECs were infected with the indicated strains of *C*. *rodentium* at a multiplicity of infection (MOI) of 100 for 3 h, as described previously [20]. Cells were counted using a hemacytometer prior to each experiment and equal numbers of cells (1–2 × 10^6^) were aliquoted into each infection condition. To determine bacterial attachment to CECs infected in suspension, cells and bacteria were passed through a Percoll gradient (40% and 60%) and CECs were collected from the top of half of the 40% gradients and overlay [62,63,64]. Cells were washed with PBS and placed on cover slips or lysed for immunofluorescence staining or immunoblot.

### Immunofluorescence staining in colon epithelial cells

Post *C*. *rodentium* infection, mouse CECs were spun down to Poly-L-Lysine-coated coverslips, fixed with 4% PFA, and stained with appropriate primary antibodies and fluorescence dye-conjugated second antibodies. Following staining of nuclei with 1 μg ml^-1^ of DAPI (Sigma-Aldrich), coverslips were mounted onto slides using Fluoro-gel with Tris Buffer (Electron Microscopy Sciences, Hatfield, PA) and examined using an Axio Observer fluorescence microscope (Zeiss, Oberkochen, Germany). The numbers of *C*. *rodentium* that attached to mouse CECs were quantified using ImageJ software (NIH, Bethesda, MD) and normalized to cell perimeter.

### *Citrobacter rodentium* infection in mice

Male C57BL/6 mice (6 to 8 weeks) purchased from the Jackson Laboratory (Bar Harbor, ME) were maintained in a specific pathogen-free facility and fed autoclaved food and water *ad libitum*. Food was withheld from the mice for 6–8 hours before they were orally inoculated with 200 μl of PBS containing 2 × 10^9^ CFU of wild-type or *ΔnleC* mutant *C*. *rodentium* or PBS alone, and euthanized at the indicated time points post infection.

### Colon tissue collection, histology, and immunofluorescence staining

After euthanizing mice, their colons were removed under aseptic conditions, washed once with ice-cold PBS, and the terminal 0.5-cm piece of the colon was frozen in optimal cutting temperature (O.C.T.) media (Tissue-Tek, Elkhart, IN) or incubated overnight in 4% PFA. 5-micron frozen sections were cut using a Microm HM 550 Cryostat (Thermo Scientific), collected on coated slides and processed for immunofluorescence staining. Frozen sections were fixed in paraformaldehyde, washed with PBS, and blocked with appropriate sera in PBS. After incubating with appropriate antibodies, sections were washed and incubated with fluorescence dye-conjugated second antibodies and 1 μg ml^-1^ of DAPI (Sigma-Aldrich). Stained sections were washed and mounted under a coverslip using Fluoro-gel with Tris Buffer (Electron Microscopy Sciences). For histological analysis, the colon tissue was embedded in paraffin and 5-micron sections were cut, collected on coated slides and processed for Hematoxylin and Eosin (H&E) staining. Stained sections were examined using an Axio Observer fluorescence microscope (Zeiss). Histopathology scores were determined in a blinded fashion using the following criteria as previously described by Qualls et al. [65]: 0, Normal tissue; Grade 1, mild inflammation was present containing mostly mononuclear cell infiltrate and little damage to the epithelia; Grade 2, inflammation greater than Grade 1 with mononuclear and polymorphonuclear infiltrate, mucin and Goblet cell depletion, and epithelium beginning to detach from basement membrane; Grade 3, inflammation and cellular infiltrate is greater than Grade 2 with cellular infiltrates reaching the submucosa, greater Goblet cell depletion, and greater epithelial disruption; Grade 4, severe inflammation containing mostly neutrophils, completely detached epithelium, and crypt destruction.

### Quantitative real-time PCR

Total RNA was isolated from colon tissues using Trizol reagent (Life Technologies) and treated with the TURBO DNA-free Kit (Life Technologies) to remove residual genomic DNA. cDNA was synthesized using qScript cDNA SuperMix Kit (Quanta Biosciences, Gaithersburg, MD) according to the manufacturer’s instructions. Gene specific products were amplified using SsoAdvanced SYBR Green Supermix (Bio-Rad Laboratories, Hercules, CA) with the following primers: *Cxcl1*-f, 5’-TGCACCCAAACCGAAGTCAT-3’; *Cxcl1*-r, 5’-TTGTCAGAA GCCAGCGTTCAC-3’; *Cxcl2*-f, 5’-CCTGCCAAGGGTTGACTTCA-3’; *Cxcl2*-r, 5’-TTCTGTCTGGGCGCA GTG-3’; *Ifng*-f, 5’-ATGAACGCTACACACTGCATC-3’; *Ifng*-r, 5’-CCATCCTTTTGCCAGTTCCTC-3’; *Il1b*-f, 5’-GAAATGCCACCTTTTGACAGTG-3’; *Il1b*-r, 5’-CTGGATGCTCTCATCAGGACA-3’; *Il22*-f, 5’-CAGAGGTAGACTTGATAACCAC-3’; *Il22*-r, 5’-GGTTATGGAAATGAAGTTACATAAGC-3’.

### Subcellular fractionation

Subcellular fractionation was performed by differential centrifugation as previously described [35]. Briefly, cells were resuspended in Buffer A (10 mM HEPES [pH 7.9], 10 mM KCl, 1.5 mM MgCl_2_, 0.1 mM EDTA, 0.5 mM DTT, 0.4% NP-40, 0.5 mM PMSF, 1 × complete protease inhibitor cocktail [Roche Applied Science]) at 4°C for 5 min. Lysates were centrifuged at 4°C, 500 × *g* for 3 min, and supernatants were collected as cytosolic fractions. Pellets were incubated in Buffer C (20 mM HEPES [pH 7.9], 420 mM NaCl, 1.5 mM MgCl_2_, 25% glycerol, 0.5 mM PMSF, 0.2 mM EDTA, 0.5 mM DTT, 1 × complete protease inhibitor cocktail) at 4°C for 10 min, followed by a centrifuge at 4°C, 13,000 × *g* for 10 min. Supernatants were collected as nuclear fractions.

### Immunoprecipitation and immunoblot

The cells were harvested and lysed on ice with 0.4 ml of lysis buffer (50 mM Tris-HCl [pH 8.0], 150 mM NaCl, 1% NP-40 and 0.5% sodium deoxycholate, 1 × complete protease inhibitor cocktail) for 10 min. The lysates were centrifuged at 10,000 × *g* at 4°C for 10 min. The protein-normalized lysates were subjected to immunoprecipitation by adding 10 mg ml^-1^ of the appropriate antibody, 30 μl of protein G-agarose (Roche Applied Science), and rotating for more than 2 h in the cold room. The precipitates were washed at least four times with cold lysis buffer followed by a separation by SDS-PAGE under reduced and denaturing conditions. The resolved protein bands were transferred onto nitrocellulose membranes (Bio-Rad Laboratories, Hercules, CA), probed as described previously [35], developed by the Super Signaling system (Thermo Scientific) according to the manufacturer’s instructions, and imaged using a FluorChem E System (Protein Simple, Santa Clara, CA).

### Luciferase reporter gene assays

Luciferase reporter gene assays were performed as previously described [18,34,40]. Briefly, cells were cotransfected with 5 × Ig κB site-driven firefly luciferase constructs and the Renilla luciferase pTKRL plasmid (ratio 10:1), together with appropriate plasmids. Cells were cultured for 18 hours, stimulated in triplicate, and analyzed using the Dual-Luciferase Kit (Promega, Madison, WI).

### Statistical analysis

All statistical analysis was performed using GraphPad Prism version 6.0 (GraphPad Software, San Diego, CA). The difference between treated and control groups were examined by unpaired Student’s *t* tests. Standard errors of means (s.e.m.) were plotted in graphs. n.s. means non-significant difference and significant differences were considered * at *p* < 0.05; ** at *p* < 0.01; and *** at *p* < 0.001.

## Supporting Information

S1 Fig The conserved Cys/Glu residues in their respective Rel domains in the mouse and the unique N-terminal 20 residues in p65/RelA are highlighted. The numbers at right show the position in the amino-acid sequence of the last residues depicted.
(TIF)

S2 Fig Whole cell lysates derived from HEK293T cells were incubated with the indicated amount of wild-type (WT) or catalytically inactive H117Y mutant His-NleC recombinant protein, followed by SDS/PAGE separation and immunoblotted for indicated proteins. The full-length p65 and cleaved p65 C-terminal fragment are indicated by filled and open triangles, respectively.
(TIF)

S3 Fig A-B. HEK293T cells (A) and HEK293T cells expressing N-terminally GFP-tagged p65 (B) were treated with 50 ng ml-1 of TNF for 30 min or the PBS vehicle control. Whole cell lysates were derived and incubated with the indicated amount of His-NleC recombinant protein, followed by SDS/PAGE separation and immunoblotted for indicated proteins. The full-length p65 and cleaved p65 C-terminal fragment are indicated by filled and open triangles, respectively.
(TIF)

S4 Fig Whole cell lysates derived from HEK293T cells expressing N- and C-terminally FLAG-tagged p50 were incubated with the indicated amount of His-NleC recombinant protein. The cleavage of FLAG-tagged p50 was immunoblotted with anti-FLAG antibody, following SDS/PAGE separation. The NleC cleavage sites in p50 are indicated by red triangles.
(TIF)

S5 Fig Whole cell lysates derived from HEK293T cells expressing FLAG-tagged RPS3 were incubated with indicated amount of His-NleC recombinant protein. The cleavage of FLAG-tagged RPS3 was immunoblotted with anti-FLAG antibody, following SDS/PAGE separation.
(TIF)

S6 Fig Various truncations of N- or C-terminally GFP-tagged p65, as indicated, were transfected into HEK293T cells. Whole cell lysates were subjected to the His-NleC cleavage assays, and immunoblotted with anti-GFP antibody for NleC-cleaved fragments. The cleaved fragments from GFP-p65 and p65^1–186^-GFP proteins were labeled by filled and open triangles, respectively.
(TIF)

S7 Fig Whole cell lysates derived from HEK293T cells expressing the indicated GFP-tagged p65 proteins were incubated with His-NleC at 4°C. Nickel beads were added to pull-down His-NleC and associated proteins. Samples were separated by SDS/PAGE, followed by immunoblot for indicated proteins.
(TIF)

S8 Fig A. Caco-2 cells were mock infected or infected with the indicated EPEC strains at 100 MOI for 1 h. Whole cell lysates were derived, separated by SDS/PAGE, and immunoblotted for indicated proteins. B. Mouse colon epithelial cells (CECs) were mock infected or infected in suspension with the indicated *C*. *rodentium* strains at 100 MOI for 3 h. Whole cell lysates were derived, separated by SDS/PAGE, and immunoblotted for indicated proteins.
(TIF)

S9 Fig Colons were harvested from C57BL/6 mice at day 10 post inoculation with the indicated *C*. *rodentium* strains and cleaned of their contents, and homogenized in PBS. Serial dilutions were performed and plated on MacConkey agar plates. Colonies were counted to determine the CFU/g of colon tissue.
(TIF)

S10 Fig Pull-down with recombinant GST or GST-RPS3 proteins with whole cell lysates derived from HEK293T cells expressing GFP-p65^1–38^, followed by Ponceau S staining and immunoblotted (IB) for GFP-p65^1–38^. (TIF)
