# Supplementary figures and images for: Metalloprotease NleC Suppresses Host NF-κB/Inflammatory Responses by Cleaving p65 and Interfering with the p65/RPS3 Interaction
Source: PLoS Pathog. 2015 Mar 10;11(3):e1004705. doi: 10.1371/journal.ppat.1004705 (PMC4355070; doi:10.1371/journal.ppat.1004705)

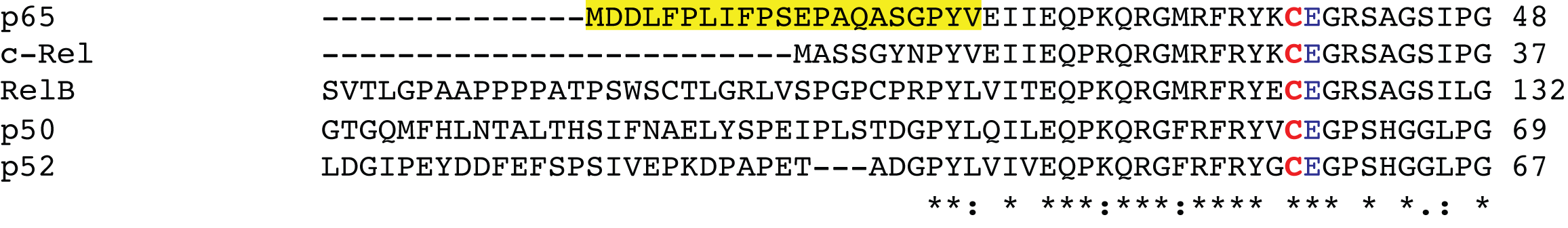

Supplement: S1 Fig — The numbers at right show the position in the amino-acid sequence of the last residues depicted. (TIF) [file ppat.1004705.s001.tif]

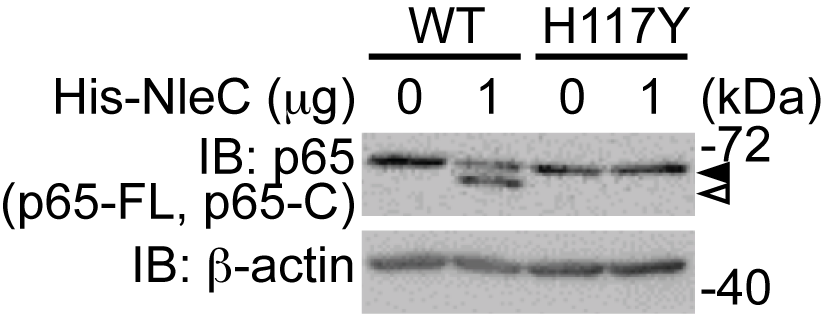

Supplement: S2 Fig — The full-length p65 and cleaved p65 C-terminal fragment are indicated by filled and open triangles, respectively. (TIF) [file ppat.1004705.s002.tif]

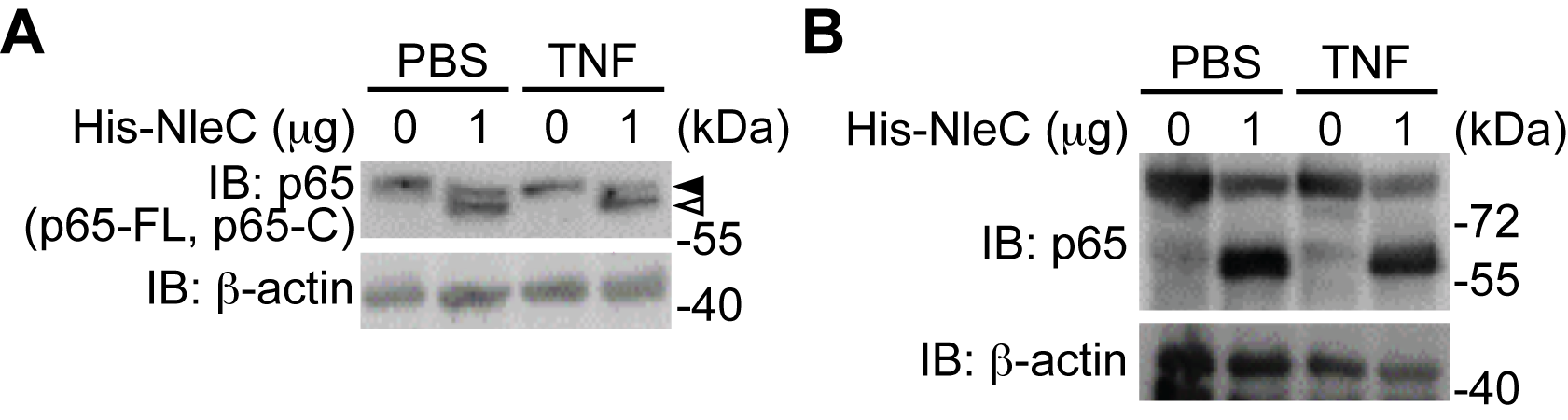

Supplement: S3 Fig — Whole cell lysates were derived and incubated with the indicated amount of His-NleC recombinant protein, followed by SDS/PAGE separation and immunoblotted for indicated proteins. The full-length p65 and cleaved p65 C-terminal fragment are indicated by filled and open triangles, respectively. (TIF) [file ppat.1004705.s003.tif]

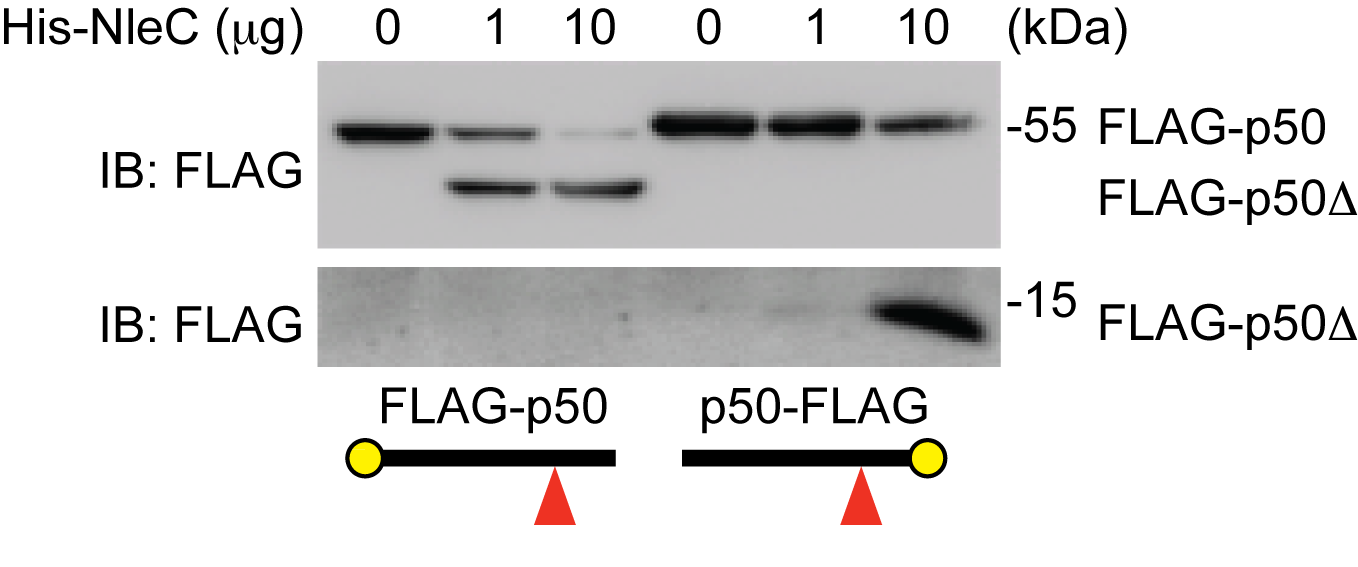

Supplement: S4 Fig — The cleavage of FLAG-tagged p50 was immunoblotted with anti-FLAG antibody, following SDS/PAGE separation. The NleC cleavage sites in p50 are indicated by red triangles. (TIF) [file ppat.1004705.s004.tif]

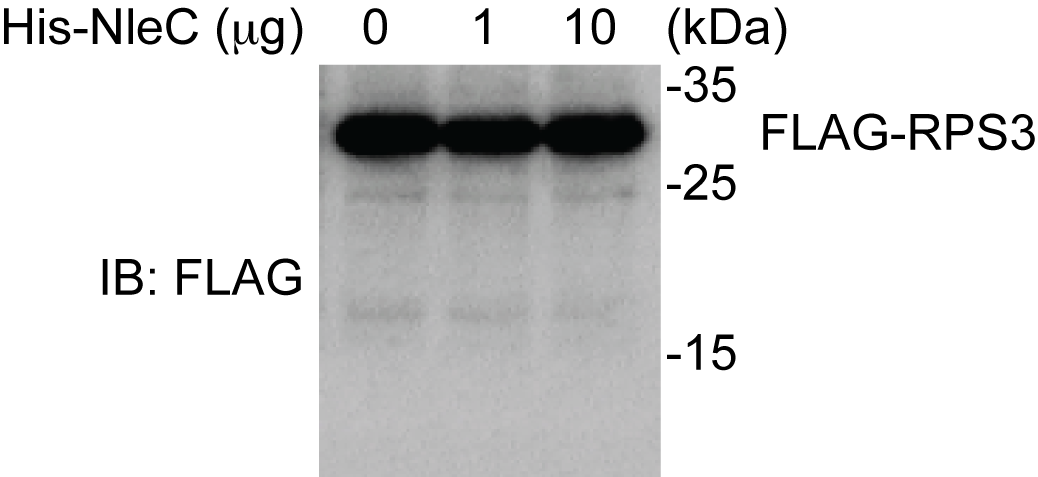

Supplement: S5 Fig — The cleavage of FLAG-tagged RPS3 was immunoblotted with anti-FLAG antibody, following SDS/PAGE separation. (TIF) [file ppat.1004705.s005.tif]

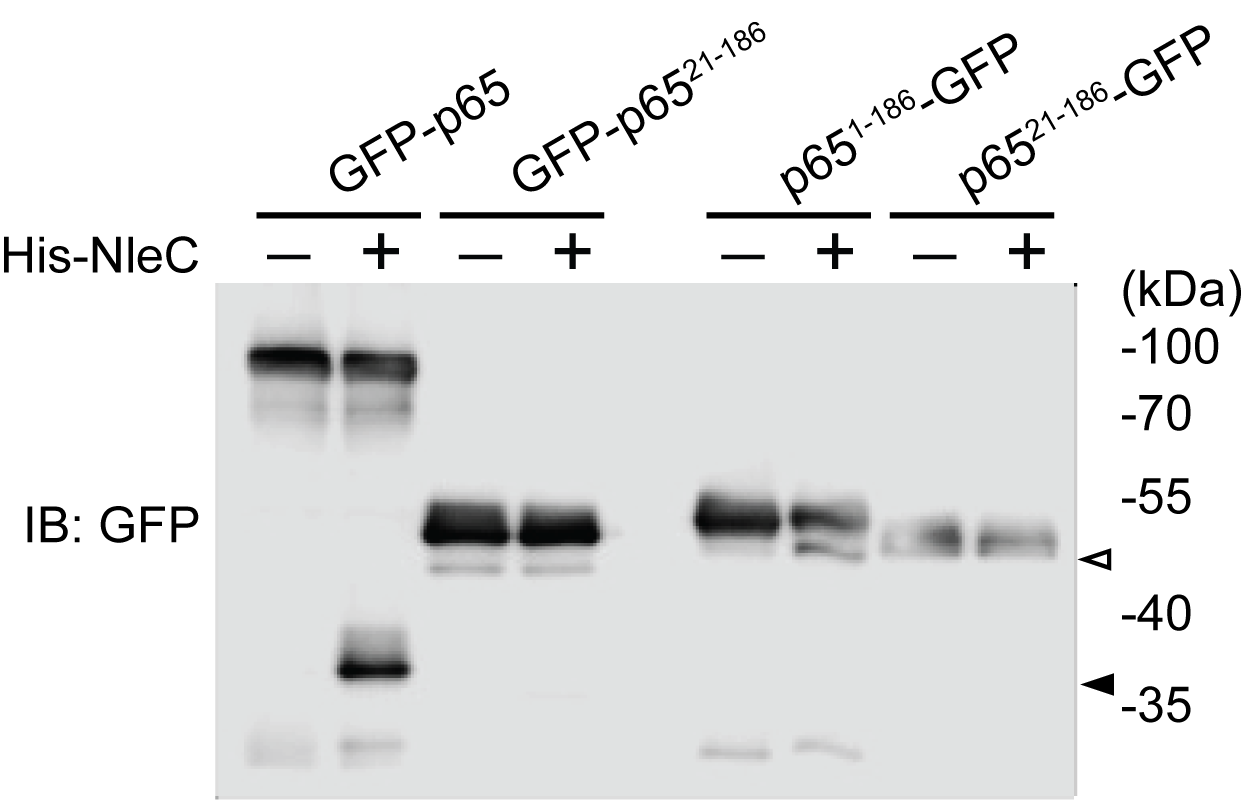

Supplement: S6 Fig — Whole cell lysates were subjected to the His-NleC cleavage assays, and immunoblotted with anti-GFP antibody for NleC-cleaved fragments. The cleaved fragments from GFP-p65 and p651–186-GFP proteins were labeled by filled and open triangles, respectively. (TIF) [file ppat.1004705.s006.tif]

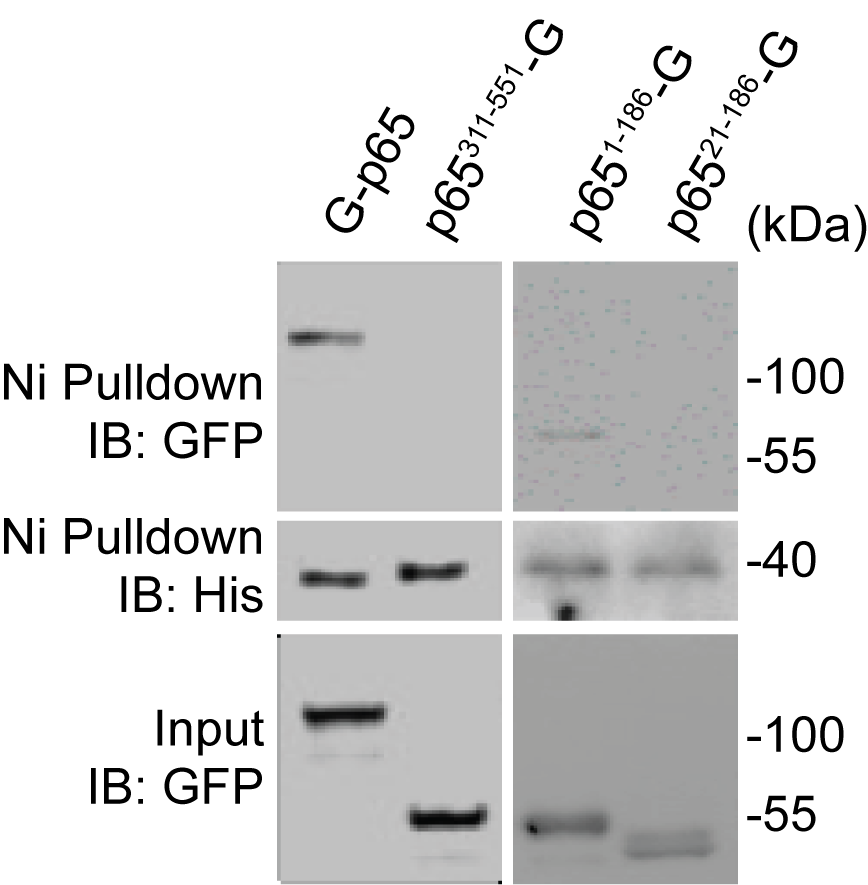

Supplement: S7 Fig — Nickel beads were added to pull-down His-NleC and associated proteins. Samples were separated by SDS/PAGE, followed by immunoblot for indicated proteins. (TIF) [file ppat.1004705.s007.tif]

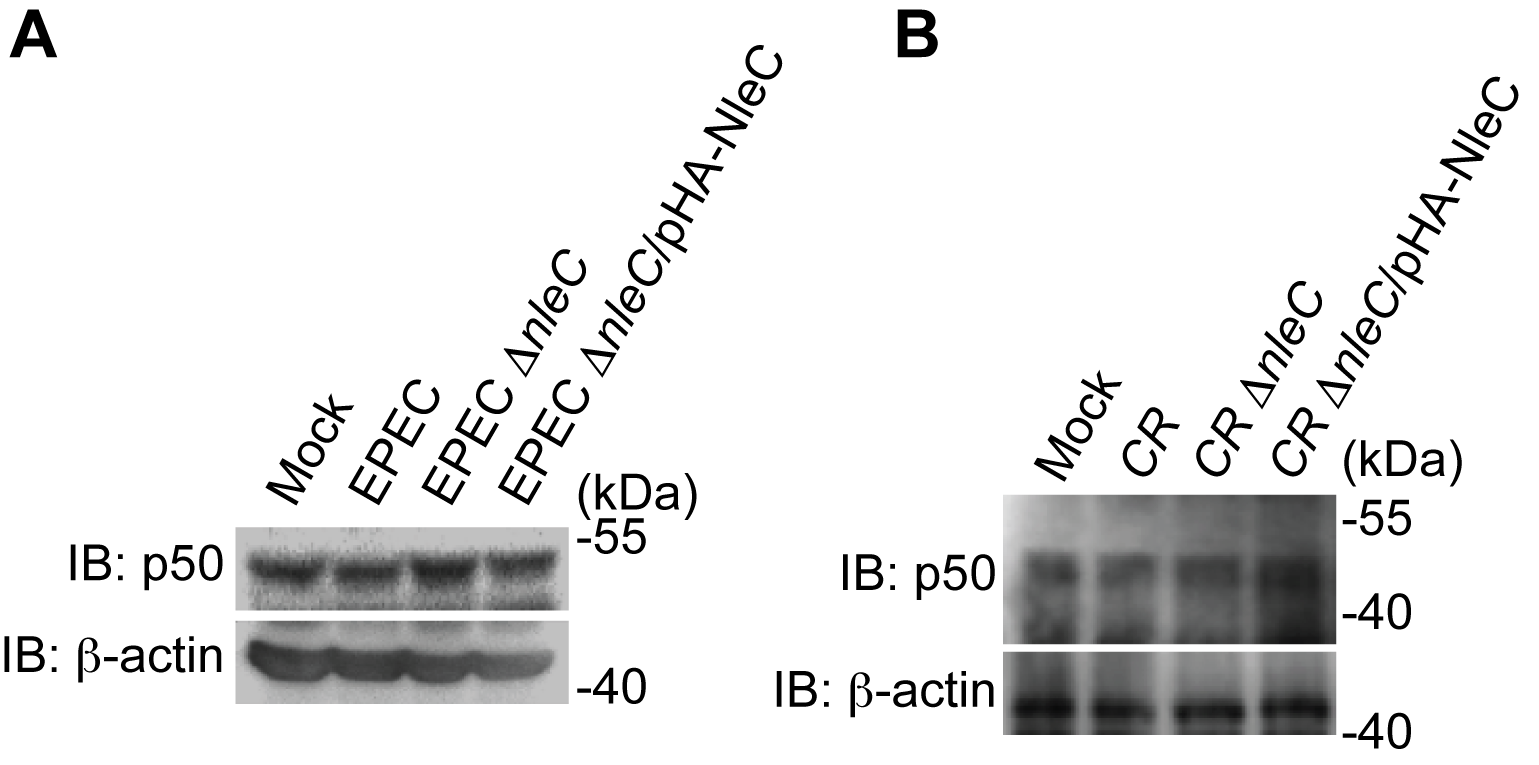

Supplement: S8 Fig — Whole cell lysates were derived, separated by SDS/PAGE, and immunoblotted for indicated proteins. (TIF) [file ppat.1004705.s008.tif]

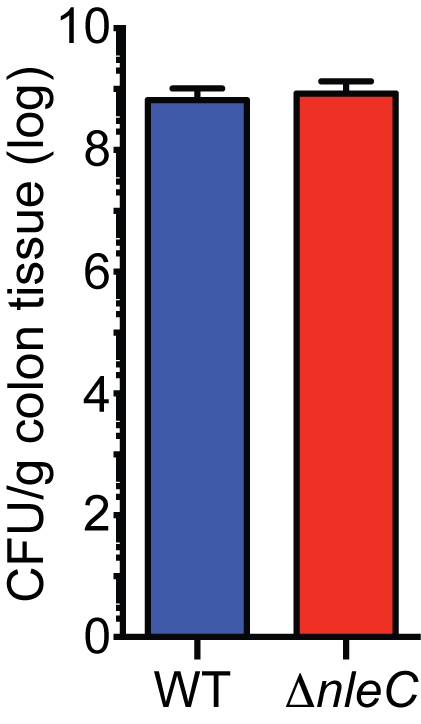

Supplement: S9 Fig — Serial dilutions were performed and plated on MacConkey agar plates. Colonies were counted to determine the CFU/g of colon tissue. (TIF) [file ppat.1004705.s009.tif]

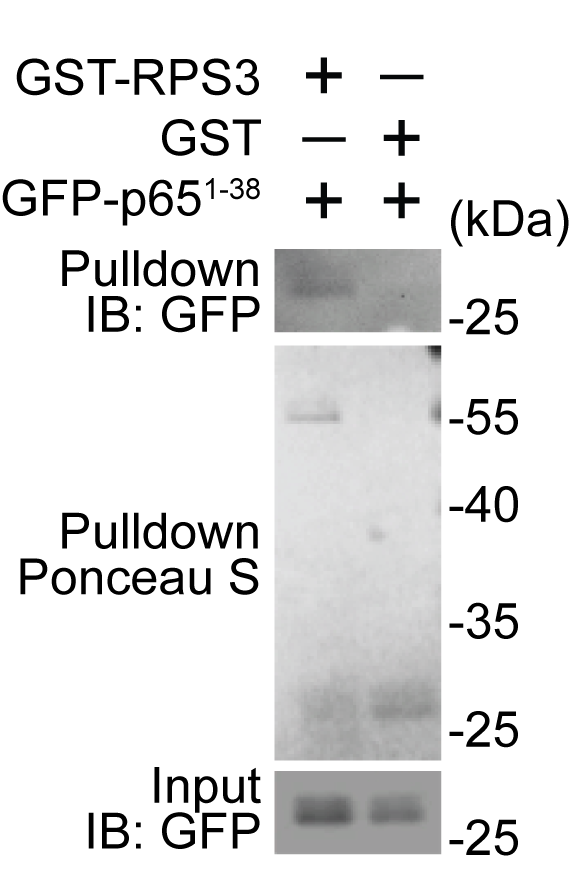

Supplement: S10 Fig — (TIF) [file ppat.1004705.s010.tif]
